# Cost-effectiveness of Paravertebral versus EPidural analgesia in Minimally invasive Esophageal resectioN (PEPMEN): an economic evaluation alongside a randomized clinical trial

**DOI:** 10.1007/s00464-026-12642-7

**Published:** 2026-03-04

**Authors:** Cezanne D. Kooij, Kirsten Opmeer, Minke L. Feenstra, Wietse J. Eshuis, Eline M. de Groot, Jeroen Hermanides, B. Feike Kingma, Suzanne S. Gisbertz, Jelle P. Ruurda, Freek Daams, Marije Marsman, Oscar F. C. van den Bosch, Werner ten Hoope, Lucas Goense, Misha D. P. Luyer, Grard A. P. Nieuwenhuijzen, Harm J. Scholten, Marc Buise, Marc J. van Det, Ewout A. Kouwenhoven, Franciscus van der Meer, Geert W. J. Frederix, Markus W. Hollmann, Edward Cheong, Miriam P. van der Meulen, Mark I. van Berge Henegouwen, Richard van Hillegersberg

**Affiliations:** 1 Department of Surgery, University Medical Center Utrecht, Heidelberglaan 100 G04.228, 3584 CX Utrecht, The Netherlands; 2 Department of Epidemiology and Health Economics, University Medical Center Utrecht, Utrecht, The Netherlands; 3 Department of Surgery, Amsterdam UMC Location University of Amsterdam, Amsterdam, The Netherlands; 4 Cancer Treatment and Quality of Life, Cancer Center Amsterdam, Amsterdam, The Netherlands; 5 Department of Anesthesiology, Amsterdam UMC Location University of Amsterdam, Amsterdam, The Netherlands; 6 Department of Anesthesiology, University Medical Center Utrecht, Utrecht, The Netherlands; 7 Department of Anesthesiology, Amsterdam UMC Location Vrije Universiteit Amsterdam, Amsterdam, The Netherlands; 8 Department of Surgery, Catharina Hospital, Eindhoven, The Netherlands; 9 Department of Anesthesiology, Catharina Hospital, Eindhoven, The Netherlands; 10 Department of Anesthesia and Pain Medicine, Maastricht University Medical Center, Maastricht, The Netherlands; 11 Department of Surgery, Hospital Group Twente, Almelo, The Netherlands; 12 Department of Anesthesiology, Hospital Group Twente, Almelo, The Netherlands; 13 Department of Upper GI, PanAsia Surgery, Singapore, Singapore

**Keywords:** Cost-effectiveness, Economic evaluation, Minimally invasive esophagectomy, Paravertebral analgesia, Epidural analgesia

## Abstract

**Background:**

Epidural analgesia has been the gold standard for pain management in minimally invasive esophagectomy (MIE), with paravertebral analgesia as a safe alternative. This cost-effectiveness analysis, conducted alongside a randomized controlled trial, evaluates the cost-effectiveness of paravertebral analgesia compared to epidural analgesia.

**Methods:**

This multicenter randomized controlled trial across four Dutch hospitals, including 192 patients, compared epidural and paravertebral analgesia in patients undergoing MIE. Cost-effectiveness was evaluated from a healthcare perspective, including in-hospital costs only, with time horizons from initial hospital stay and 90 days postoperatively. Procedural costs were calculated with a bottom-up approach, and analgesia costs with data from the electronic case report form (eCRF). Other hospital costs were calculated using insurance claim data from hospital registries. Effectiveness was displayed as Quality of Recovery (QoR-40) scores. Bootstrapping was used to estimate uncertainty.

**Results:**

Mean initial surgery costs were €10,469 for the epidural and €10,051 for the paravertebral group, primarily due to a shorter mean operating room time (mean difference 19 min; 95% CI − 7 to 45). Mean postoperative (day 1–3) medication costs were €116 and €125, respectively. During the initial hospital stay, mean total in-hospital costs for uncomplicated patients were €8557 and €8646, and for complicated patients €28,244 and €28,387, respectively. When excluding complications, bootstrapping showed that 47.9% of iterations had lower costs and lower effectiveness for paravertebral analgesia, with the other iterations across all other quadrants.

**Conclusions:**

Cost differences between paravertebral and epidural analgesia were minimal, with cost-effectiveness primarily influenced by the slightly lower effectiveness of paravertebral analgesia.

**Supplementary Information:**

The online version contains supplementary material available at 10.1007/s00464-026-12642-7.

Esophageal cancer surgery consists of esophagectomy with radical lymphadenectomy, which is a complex procedure involving both thoracic and abdominal surgery [1]. Despite implementation of minimally invasive surgical techniques, effective pain management remains essential for optimizing patient comfort, enhancing recovery and shortening hospitalization [2]. Pain control for esophagectomy patients has historically relied on multimodal intravenous analgesia. However, in recent decades, regional anesthesia techniques, including epidural and paravertebral analgesia, have become the standard of care [3]. Epidural analgesia has traditionally been the preferred method; however, its superiority over other modalities remains debated [3–5]. The advent of minimally invasive esophagectomy (MIE) has further increased the interest in alternative regional analgesia techniques [3]. Additionally, challenges associated with epidural analgesia, such as failure of placement or dislocation, complications and adverse effects like hypotension, urinary retention and reduced mobility, highlight the need to explore alternative options [5–9]. Therefore, the recent Paravertebral versus EPidural Analgesia in Minimally Invasive Esophageal resectioN (PEPMEN) trial compared paravertebral to epidural analgesia in MIE. This trial demonstrated the safety and applicability of paravertebral analgesia as a viable alternative to epidural analgesia [10]. For implementation in practice, it is important to evaluate not only clinical feasibility and usability but also the costs associated with each technique. It was hypothesized that if paravertebral analgesia would result in more effective pain management after surgery, it could also result in lower costs, primarily due to an anticipated shorter stay in the intensive care unit (ICU) or post-anesthesia care unit (PACU).

As part of the trial, a cost-effectiveness analysis was intended to compare paravertebral analgesia with epidural analgesia. This analysis aims to determine whether paravertebral analgesia provides a cost-effective alternative compared to epidural analgesia in MIE.

## Materials and methods

A cost-effectiveness study was performed alongside the PEPMEN-trial, comparing the costs and effects associated with paravertebral analgesia versus epidural analgesia in MIE. Effectiveness was displayed as Quality of Recovery-40 (QoR-40) scores at postoperative day (POD) 3 [11]. A healthcare perspective was used, including in-hospital costs only, with different time horizons varying from initial hospital stay after MIE to the first 90 days after surgery. This approach was chosen because the main cost differences between analgesic strategies are expected to occur during hospital stay and early postoperative period. Due to the short time horizon, discounting was redundant [12].

### PEPMEN-trial

The PEPMEN-trial was a multicenter randomized controlled superiority trial comparing epidural analgesia with paravertebral analgesia for MIE in four Dutch hospitals (UMC Utrecht; Amsterdam UMC; Catharina Hospital Eindhoven; ZGT Almelo). The protocol was registered in the Dutch Trial register (NL8037) and published at trial initiation [11]. In short, patients undergoing elective MIE with two-field lymphadenectomy, gastric conduit reconstruction, and intrathoracic anastomosis were eligible. After written informed consent, patients were randomized to paravertebral or epidural analgesia, stratified per hospital. Results were recently published elsewhere [10]. From December 2019 to February 2023, 192 patients were included, with 98 assigned to paravertebral analgesia and 94 to epidural analgesia (Supplementary Fig. S1). The primary outcome, the QoR-40 score on POD 3, showed no significant difference between the epidural and paravertebral group in both intention-to-treat (168.8 vs. 165.1, *P* = 0.268) and per-protocol analyses (168.8 vs. 165.2, *P* = 0.273) [10].

### Ethics

Ethical approval for this study (Ethical Committee Number 19/588) was provided by the Ethical Committee of the UMC Utrecht, Utrecht, The Netherlands (Chairperson: Dr. G. van Thiel) on November 19, 2019.

### Resource use and related costs

The following cost items (in euros) were included: unit costs of surgery, medication costs and in-hospital healthcare costs. Unit costs of MIE were calculated with a bottom-up approach, medication was registered in the electronic case report form (eCRF), healthcare resource use was calculated using patient-level care activities from claim data of all participating hospitals and unit costs from the Dutch costing guideline and Dutch Hospital Authority (NZa)-rates. [12, 13].

#### Unit costs of MIE

Unit costs of surgery were calculated with a bottom-up approach for both MIE with epidural and paravertebral analgesia. The unit costs included costs of the operating room (OR) (including the room itself, personnel, and overhead), laparoscopic equipment costs per surgical procedure, disposable epidural and/or paravertebral materials, and medication intra-operatively.

OR costs were estimated by multiplying each patient’s time in the OR-anesthesia start to end (or, if unavailable, until surgery end)-by the minute price of the OR, calculated by Bolkenstein et al. [14, 15] A sensitivity analysis was conducted using more recent cost estimates from the guidelines for health economic analysis in the Netherlands. [12].

Laparoscopic equipment costs per surgical procedure were calculated based on previous calculations by Van der Veen et al. (Supplementary Table S1) [16]. Disposables and corresponding costs for epidural and/or paravertebral catheter placement were provided by each hospital’s anesthesiology department (Supplementary Table S2).

#### Medication costs

Doses of vasopressor drugs, opioids and catheter-delivered medication were recorded per patient during surgery and on POD 1–3 using the eCRF. Medication costs were calculated using open-source pricing data [17, 18]. Flacon prices were recalculated to mg(ug)/ml prices. With these parameters, medication daily costs per patient and daily average costs were calculated for vasopressors, opioids and catheter medication.

#### In-hospital healthcare resource use and costs

In-hospital resource use was collected using claim data from all participating hospital registries. Claim data were missing for two patients; these patients were excluded from the analysis. Costs per patient were calculated by multiplying healthcare resource use by the unit costs of each procedure. Unit costs were based on prices from the Dutch Healthcare authority and the Dutch guideline on costing research in healthcare [12, 13].

### Effect measure

In the PEPMEN-trial the EuroQol 5-dimension (EQ-5D-5L) questionnaire was carried out preoperatively, and 3 and 6 months postoperatively. Although the EQ-5D-5L is commonly used to calculate quality-adjusted life-years (QALYs) in cost-utility analyses [19, 20]. It captures multiple aspects beyond the analgesic effect. Given that analgesic technique primarily influences short-term postoperative recovery after MIE, we selected the QoR-40 questionnaire as the most appropriate measure to assess the impact of analgesia on postoperative recovery. This 40-item questionnaire (score range 40–200) assesses five dimensions to evaluate early postoperative health [21]. Patients were asked to complete the QoR-40 pre-operative and on POD 1–3. The score on POD 3 was the endpoint.

### Scenario analyses

Scenario analyses provide insights into resource use and costs across subgroups and different timeframes. Two time horizons were selected: from POD 1 to the end of initial hospital stay and from POD 1 till POD 90. The first time horizon highlights the effect of paravertebral or epidural analgesia on costs in the immediate postoperative period. The second time frame was chosen based on the expectation that, starting from three months postoperatively, random variation in disease progression and potential start of anti-cancer treatment will overshadow the effects of analgesia costs. Extending the time horizon beyond three months postoperatively is expected to reflect heterogeneity in (impact of) subsequent anti-cancer treatment rather than differences in analgesia use. While out-of-hospital costs are not captured, the 90-day postoperative time frame provides partial insight into post-discharge resource use, including readmissions, post-discharge visits, and complications occurring within this period.

Subgroups were defined based on the occurrence of complications within 30 days after surgery, as complications are a major driver of postoperative resource use and costs. Patients were divided into two subgroups: those with no complications and those with complications. It is likely that complications were independent of the type of analgesia employed. Complications included anastomotic leakage, gastric tube necrosis, stricture of the anastomosis, pulmonary complications, cardiac complications, intra-abdominal hemorrhage, ileus, intra-abdominal abscess, sepsis, wound infection, urinary tract infection and tracheoesophageal fistula.

### Cost-effectiveness analysis

Statistical analysis was performed with the use of RStudio (R foundation of Statistical Computing, version 2024.04.0+735). Missing QoR-40-values and missing doses opioid, vasopressors and catheter medication were imputed with the multiple imputing with the R MICE package, 20 imputed data sets were created [14]. Before imputation the total QoR-40 scores per patient were determined by taking the sum of all items, with exception for preoperative scores where question 16, 17 and 18 were not taken into consideration.

Thereafter, a parcel summary score was taken for each day to use in the imputation [22]. The following predictors were used; treatment group, sex, BMI, age, hospital, american society of anesthesiologists (ASA) score, medical history parameters (cardiac, pulmonary, cerebrovascular, liver disease, diabetes mellitus and hypertension). Apart from two patients with missing data on start and end of surgery and two patients missing in cost data, data on healthcare resource use from all four hospitals were complete.

Total costs and QoR-40 scores were estimated per patient and mean values compared for epidural and paravertebral analgesia. To estimate uncertainty in costs and QoR-40 outcomes, bootstrapping was used with 2000 iterations [12]. For all 20 imputed data sets. [14]. Subsequently, a cost-effectiveness plane was created for each scenario to show the statistical uncertainty. Each dot in the plane represents the cost differences and effect differences of one bootstrap iteration. To assess the influence of extreme cost values, a subgroup analysis was performed in patients without postoperative complications. Analyses were performed in accordance with intention-to-treat, and impacts were assessed uniformly across all individuals in the study, including those who did not receive the assigned epidural or paravertebral catheter or had premature catheter removal, with no specific adjustments for priority populations or sociodemographic characteristics.

## Results

### Unit costs of MIE

The mean initial surgery costs were estimated to be €10,469 for the epidural and €10,051 for the paravertebral group (Table 1). Most of these costs were attributed to the per-minute price of the surgery. With a mean OR time of 467 min (SD 87 min, 95% CI 449–484 min) for the epidural and 448 min (SD 92 min, 95% CI 431–467 min) for the paravertebral group, this resulted in €10,277 and €9862, respectively. The disposable material costs for epidural catheter placement were estimated to be €4.50 higher than those for paravertebral catheter placement (Table 1, Supplementary Table S2). Additionally, six patients in the epidural group ultimately did not receive an epidural analgesia catheter. In four of these patients, the epidural attempt was unsuccessful, leading to intraoperative placement of a paravertebral catheter, resulting in the use of both sets of analgesia materials. In one patient, the epidural catheter placement failed (after use of the epidural materials), and the patient was treated with multimodal intravenous analgesia. For the remaining patient, only the paravertebral set was used, as it was clear before attempting that epidural catheter placement would not be successful. Regarding the intraoperative medication costs, the total of expenses for analgesia catheter medication, opioids and, if needed, vasopressors were slightly higher in the paravertebral group (mean difference €1.63).

**Table 1:** Unit costs of minimally invasive esophagectomy with epidural or paravertebral analgesia, as calculated with a bottom-up approach

Units	Costs per unit	Epidural (*n* = 94)		Paravertebral (*n* = 98)	
		Mean no. of units	Mean cost, €	Mean no. of units	Mean cost, €
Minute price of operation (including operation room, personnel and overhead)^*^^	22.00	467	10,277	448	9,862
Laparoscopic equipment costs per surgical procedure^#^	102.53	1	102.23	1	102.23
Disposable epidural/paravertebral materials^**^	Variable	94^&^	30.13	98	25.63
Medication intraoperatively	Variable				
Vasopressor medication		92	18.63	96	12.68
Opioids		93	28.35	98	38.60
Analgesia catheter costs		75^!,&^	12.71	46^!^	10.04
Total costs	NA	NA	10,469.05	NA	10,051.18

^^^Based on Cost-effectiveness analysis of a multicenter randomized clinical trial comparing surgery with conservative management for recurrent and ongoing diverticulitis (DIRECT trial)–Bolkenstein et al. https://doi.org/10.1002/bjs.11024 [as used in LOGICA economic evaluation]

*Operation time: start anesthesia (including epidural placement) until end operating time, 2 cases missing (no operating times at all) (start anesthesia until end anesthesia is missing in 16 cases–end anesthesia can also occur outside operating room)

^#^Details on the calculations of the laparoscopic equipment costs per surgical procedure are provided in Supplementary Table S1

**Details on the calculations of the disposable costs per participating center are provided in Supplementary Table S2

!Data on drip rate is missing in 13 epidural cases and 52 paravertebral cases (no drip rate at all, or no difference in drip rate specified)

^&^Six patients did eventually not receive an epidural catheter, of which 4 patients received a paravertebral catheter of which the drip rate and time of placement are unknown

### Medication costs

The mean (imputed) post-operative medication costs in the epidural and paravertebral group were €39.62 and €40.03 on POD 1, €38.08 and €44.89 on POD 2, and €38.37 and €40.17 on POD 3, respectively (Table 2). In total, the mean imputed costs in the epidural group were €116.07 and €125.09 in the paravertebral group. On POD 2, the cost difference was primarily due to opioid expenses (€2.86 in the epidural and €12.88 in the paravertebral group), while on POD 3, the difference was mainly attributed to medication costs for analgesia catheter (€23.17 in the epidural and €32.69 in the paravertebral group).

**Table 2:** Medication costs of minimally invasive esophagectomy with epidural or paravertebral analgesia, as calculated with a bottom-up approach

Units	Costs per unit	Epidural (*n* = 94)			Paravertebral (*n* = 98)		
		N^^^	Known^#^	Mean cost, €*	N^^^	Known^#^	Mean cost, €^*^
Medication day 1	Variable						
Vasopressor medication		37	31	2.25	13	11	1.54
Opioids		68	61	4.38	93	85	8.08
Analgesia catheter		92	90	34.30	98	87	32.69
Total		94	84	37.12	98	82	38.24
Imputed total		94	94	39.62	98	98	40.03
Medication day 2	Variable						
Vasopressor medication		8	8	4.58	4	4	2.33
Opioids		69	67	2.86	94	90	12.88
Analgesia catheter		82	82	32.15	83	82	32.69
Total		94	82	36.42	98	89	49.33
Imputed total		94	94	38.08	98	98	44.89
Medication day 3	Variable						
Vasopressor medication		4	3	3.19	4	4	6.73
Opioids		74	71	4.89	83	78	8.07
Analgesia catheter		66	66	23.17	73	48	32.69
Total		98	66	34.01	98	70	57.10
Imputed total		94	94	38.37	98	98	40.17
Total costs of imputed total	NA	NA	NA	116.07	NA	NA	125.09

^Number of patients who received (type of) medication;

^#^Number of patients who received (type of) medication without missing values in dose;

*Mean costs are calculated based on number of patients that actually received medication without missing values;

*NA* Not applicable

### In-hospital costs

The mean total in-hospital costs during initial in-hospital stay were €19,657 for the epidural and €19,134 for the paravertebral group. In patients with an uncomplicated postoperative course, mean total in-hospital costs during the initial hospital stay were €8557 for the epidural and €8646 for the paravertebral group (Table 3). In patients with a complicated postoperative course, the mean total in-hospital costs during the initial hospital stay were €28,244 for the epidural and €28,387 for the paravertebral group, with ICU and admission days as the main contributor of costs (Table 3).

**Table 3:** Number of procedures, costs and patients undergoing MIE with either epidural or paravertebral analgesia from postoperative day 1 till the end of initial hospital stay

Type of care	Epidural						Paravertebral					
	No complications			Complications			No complications			Complications		
	No. of individuals	Mean no. of procedures	Mean cost, €^!^	No. of individuals	Mean no. of procedures	Mean cost, €	No. of individuals	Mean no. of procedures	Mean cost, €	No. of individuals	Mean no. of procedures	Mean cost, €
Admissions^#^												
ICU^	18	0.5	1771	28	3.4	11,750	23	0.5	1767	25	4.2	14,549
Hospital stay	41	7.2	5303	51	13.3	9757	45	6.9	5093	49	10.5	7691
Diagnostics												
Endoscopy	0	0	0	12	0.6	307	2	0.1	10	17	1.0	300
Imaging	41	4.8	436	52	10.7	1135	45	5.2	486	51	11.7	1416
Laboratory	41	58.2	291	52	207.7	1262	45	58.1	292	50	198.6	1298
Other diagnostics	0	0	0	9	0.3	123	1	0.02	3	8	0.3	107
Consultations												
ER	0	0	0	0	0	0	0	0	0	0	0	0
Policlinic	0	0	0	1	0.02	3	1	0.04	6	0	0	0
Paramedic	37	9.2	342	44	16.3	619	39	7.6	286	43	15.0	566
Anesthesia	0	0	0	9	0.5	321	0	0	0	6	0.3	145
Other	0	0	0	3	0.1	14	0	0	0	3	0.1	15
Totals												
Total^@^	41	83.6	8557	53	290.5	28,244	47	82.4	8646	51	279.5	28,387

Patients receiving epidural or paravertebral analgesia are further divided in patients with no complications or complications. Number of patients, procedures and costs are subdivided into admission costs, diagnostic costs, costs of consultations and total costs

^ICU days were registered for 94 patients of the 192 patients in the PEPMEN trial during the initial hospital stay

^!^The mean number of procedures and mean costs are not linearly related, because different procedures have different costs

^#^Mean number of procedures under admissions represents the mean number of days

^@^Total costs also include expenses that fall outside the categories of admission, diagnostics, or consultation. These additional costs are not listed in the table

**Fig. 1:**
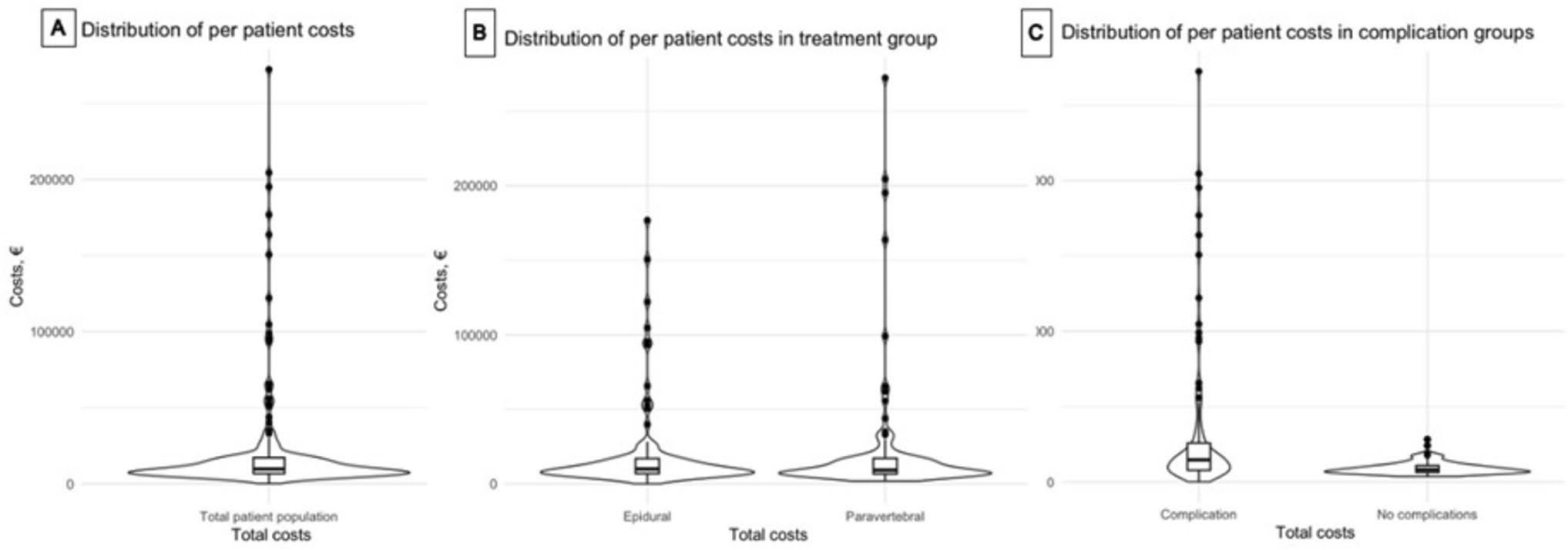
Distribution of per patient costs made visible in plots **A** Distribution of per-patient total costs during the first 3 months post-operative for all patients undergoing MIE. **B** Distribution of per-patient total costs during the first 3 months post-operative divided into two groups: patients receiving epidural analgesia and patients with paravertebral analgesia during MIE. **C** Distribution of per patient total costs of first 3 months post-operative segregated into two groups: patients with complications and patients without complications. The body of the violin plot represents the estimated density of the data: *y*-axis represents costs and the *x*-axis corresponding number of individuals (The broader the body, the more individuals). The black dots mark individual outliers. A box plot is shown in the body of the violin blot. The box plot displays the minimum and maximum excluding outliers (central black line), median (horizontal line) and IQRs (box) and the minimum

During the first three months postoperative, mean total in-hospital costs were €26,521 in the epidural and €26,178 in the paravertebral group. In patients with an uncomplicated postoperative course three months post-surgery, mean total in-hospital costs were €11,442 in the epidural and €11,762 in the paravertebral group (Table 4). In patients with a complicated postoperative course, the mean total in-hospital costs up to 3 months post-surgery were €38,186 for the epidural and €38,899 for the paravertebral group, also with ICU and admission days being the primary cost drivers (Table 4). Figure 1 Notably, five patients in each group required ICU stays exceeding ten days, ranging from 13 to 32 days in the epidural group and 12 to 65 days in the paravertebral group. (Fig. 1A) The longest stays were observed in the paravertebral group, and ICU-(re)admissions in these cases were attributed to complications from esophagectomy (anastomotic leakage, pulmonary complications, cardiac complications, neurological complications and/or sepsis). Further details on admission costs and length of hospital stay are provided in Tables 3 and 4. The distribution of costs in the epidural and paravertebral group are similar, with more extreme outliers observed in the paravertebral group (Fig. 1B). The violin plot shows that the outliers in both the epidural and paravertebral groups are predominantly patients with complications (Fig. 1C).

**Table 4:** Number of procedures, costs and patients undergoing MIE with either epidural or paravertebral analgesia from postoperative day 1 till postoperative day 90

Type of care	Epidural						Paravertebral					
	No complications			Complications			No complications			Complications		
	No. of individuals	Mean no. of procedures	Mean cost, €^!^	No. of individuals	Mean no. of procedures	Mean cost, €	No. of individuals	Mean no. of procedures	Mean cost, €	No. of individuals	Mean no. of procedures	Mean cost, €
Admissions^#^												
ICU^	18	0.5	1771	30	4.1	14.173	23	0.5	1,767	26	5.1	17,543
Hospital stay	41	8.0	5877	51	16.5	12,141	45	7.4	5,452	49	14.6	10,731
Diagnostics												
Endoscopy	10	0.7	315	23	1.8	1.235	8	0.4	190	24	2.2	721
Imaging	41	6.2	622	52	15.3	1708	45	6.7	712	51	14.3	1,729
Laboratory	41	73.8	371	52	267.7	1581	45	80.3	402	51	259.5	1,680
Other diagnostics	1	0.02	6	14	0.5	160	2	0.04	5	12	0.5	177
Consultations												
ER	8	0.2	72	19	0.6	167	8	0.3	78	15	0.6	167
Policlinic	35	2.0	274	46	2.3	315	40	2.5	338	45	2.5	338
Paramedic	38	20.0	731	44	26.9	1,001	39	17.1	623	47	28.8	1065
Anesthesia	1	0.02	10	18	1.1	595	3	0.09	15	8	0.8	537
Other	0	0	0	4	0.08	19	0	0	0	4	0.08	19
Totals												
Total^@^	41	121.8	11,442	53	400.0	38,186	47	130.1	11,762	51	383.7	38,899

Patients receiving epidural or paravertebral analgesia are further divided in patients with no complications or complications. Number of patients, procedures and costs are subdivided into admission costs, diagnostic costs, costs of consultations and total costs;

^ICU days were registered for 97 patients of the 192 patients in the PEPMEN trial in the first 90 days postoperative

^!^The mean number of procedures and mean costs are not linearly related, because different procedures have different costs

^#^Mean number of procedures under admissions represents the mean number of days

^@^Total costs also include expenses that fall outside the categories of admission, diagnostics, or consultation. These additional costs are not listed in the table

*ICU* intensive care unit; *ER* Emergency room

### Cost-effectiveness analysis

**Fig. 2:**
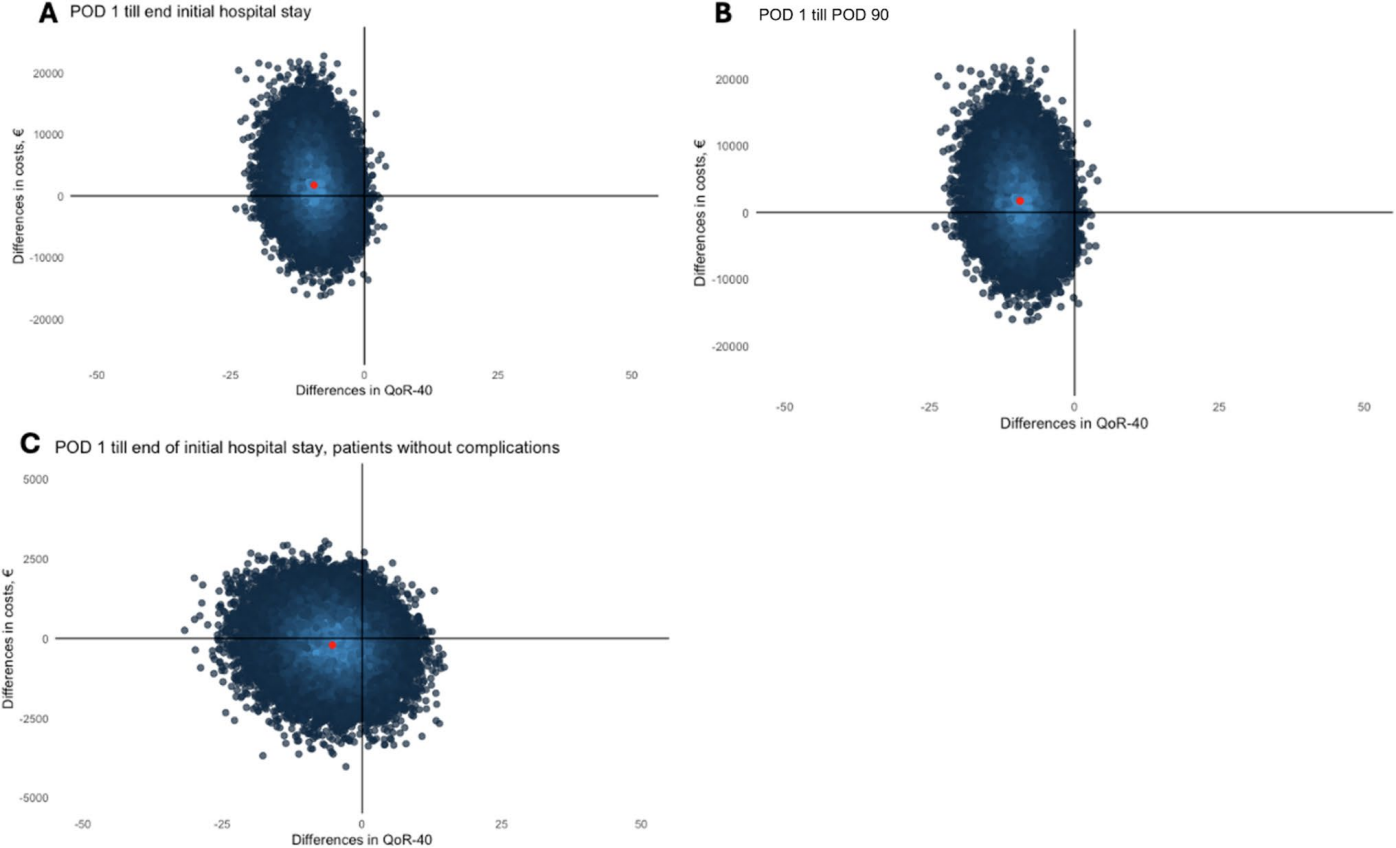
Cost-effectiveness planes costs and QoR-40 scores for the epidural group compared to the paravertebral group across 2,000 bootstrap iterations are shown in cost-effectiveness planes. **A** Cost-effectiveness plane for the initial hospital stay. **B** Cost-effectiveness plane from postoperative day 1 to 3 months post-surgery. **C** Cost-effectiveness plane for the initial hospital stay, including only patients without complications. Each dot represents the difference in cost and QoR-40 score between epidural and paravertebral analgesia for a single bootstrap iteration. Iterations in the upper left quadrant indicate higher costs and lower QoR-40 scores for the paravertebral group, the upper right quadrant shows higher costs and higher QoR-40 scores for the paravertebral group, the lower right quadrant represents lower costs and higher QoR-40 scores for the paravertebral group, and the lower-left quadrant shows lower costs and lower QoR-40 scores for the paravertebral group. The shade of blue indicates overlapping dots, with light blue representing the highest density

Figure 2 displays the outcomes of bootstrap iterations across all scenarios in cost-effectiveness planes, representing the uncertainty in cost and QoR-40 differences between the treatment groups.

In the first scenario including all patients until the end of initial hospital stay, the mean of all iterations lies just in the upper left quadrant, meaning that paravertebral analgesia has slightly higher costs and lower QoR-40 scores. Most iterations are in this quadrant (62.1%), followed by 37.7% in the bottom left quadrant, indicating lower costs and lower QoR-40 scores in the paravertebral group (Fig. 2A). A percentage of 0.1% is in the upper right quadrant and 0.1% is in the bottom right quadrant, indicating few cost-effective scenarios for the paravertebral group.

In the second scenario, including all patients up to POD 90, the mean also lies within the upper left quadrant, indicating slightly higher costs and lower QoR-40 scores in the paravertebral group. Again, two-thirds of all iterations are in this quadrant (63.3%), while 36.6% are in the bottom left quadrant (Fig. 2B). The remaining iterations are in the upper right (0.1%) and the bottom right (0.1%) quadrants.

The third scenario focused on patients without complications during the initial hospital stay. The mean lies in the bottom left quadrant, indicating that paravertebral analgesia is associated with lower costs and QoR-40 scores. Iterations are primarily spread over the left quadrants, with 35.0% in the upper left, 47.9% in the bottom left, 11.8% in the lower right and 5.4% in the upper right quadrant (Fig. 2C).

A secondary sensitivity analysis with the minute price of the OR, as stated in the guidelines for health economic analysis in the Netherlands [13], is provided in Supplementary Figure S2, demonstrating results similar to those of the scenarios depicted in Fig. 2.

## Discussion

This study compared the costs and cost-effectiveness of epidural and paravertebral analgesia in patients undergoing MIE for esophageal cancer alongside the multicenter PEPMEN-trial. Our detailed bottom-up calculation identified small cost-savings (€418) of the mean initial surgery costs for the paravertebral (€10,051) compared to the epidural group (€10,469), which was primarily due to a non-significant shorter OR time (mean difference 19 min, 95% CI −7 to 45). Cost differences in disposables and medication were minimal (mean difference €4.50 and €9.02). Among patients with an uncomplicated postoperative course, mean costs during primary hospital stay were €8,557 in the epidural and €8646 in the paravertebral group. In this uncomplicated scenario, 47.9% of bootstrap iterations fell in the lower-left quadrant of the cost-effectiveness plane, indicating lower costs and lower QoR-40 scores for paravertebral analgesia. However, when the entire cohort was analyzed without stratification for complications, the point estimate was located in the upper left quadrant, suggesting slightly higher costs and lower QoR-40 scores for the paravertebral group.

This study provides a secondary analysis of the costs and cost-effectiveness of epidural versus paravertebral analgesia in MIE, building upon the primary study, which was designed to detect a clinically relevant difference in QoR-40 on POD 3 but not powered to detect differences in variables like OR time [11]. Although the magnitude of the difference in OR time was as anticipated, the variance was too large to achieve statistical significance. OR time is a relevant cost driver contributing to the total surgery cost differences; however, this analysis does not support a causal relationship with the analgesic technique. For the minute price of the operation, we used the values provided by Bolkenstein et al. [15]. Based on our experience with similar analyses in previous studies [16]. Additionally, we conducted a secondary sensitivity analysis using the minute price of the OR as specified in the guidelines for health economic analysis in the Netherlands, which reflect more recently published costs [12]. This secondary sensitivity analysis was intended to determine whether updated cost estimates revealed differences not observed in the primary analysis. However, the results aligned with those of the primary analysis.

Disposable costs were higher for epidural analgesia (mean difference €4.50), with variations observed among participating centers (Supplementary Table S2), some using pre-packaged epidural sets while others opting for individually selected items for paravertebral catheter placement. Furthermore, in four epidural cases, catheter placement was unsuccessful, requiring subsequent paravertebral catheter was placement and incurring costs for both procedures in this intention-to-treat analysis. Regarding medication costs, intraoperative vasopressor and analgesia catheter medication costs were higher in the epidural group, but opioid costs were higher in the paravertebral group, resulting in slightly higher overall intraoperative medication costs for paravertebral group (mean difference €1.63). Similarly, medication costs during POD 1–3 were also higher in the paravertebral group (mean difference €9.02). Despite the variations in disposable and medication costs, the differences remained minimal, as was anticipated.

The cost difference between the epidural and paravertebral groups in complicated cases up to three months postoperatively was notable but relatively small (mean difference €713) compared to the overall in-hospital costs of this procedure. This difference was primarily driven by higher ICU expenses in the paravertebral group (€17,543), whereas the epidural group had lower costs (€14,173). Consequently, our hypothesis that the paravertebral group would have lower costs due to shorter ICU stay was not supported. The complications necessitating ICU (re)admission are not expected to be related to the investigated interventions and we believe this was most likely due to random chance, while the occurrence of postoperative complications, specifically anastomotic leakage and pulmonary complications, did not differ significantly between groups [10].

When interpreting the ICU-related costs, it is important to recognize that approximately half of patients in both the epidural (48.9%; 46 of 94 patients) and the paravertebral group (49.0%; 48 of 98 patients) were admitted to the ICU postoperatively. ICU admission and the associated costs may result from either routine postoperative ICU admission or from ICU stays due to complications. Notably, ICU admission protocols varied across the participating centers, which could have influenced cost comparisons. In some centers, patients were routinely admitted to the ICU postoperatively, while in others, patients were initially admitted to the PACU and only transferred to the ICU after one night if clinically indicated. Therefore, the presence or absence of ICU admissions and related costs should be interpreted with these protocol differences in mind.

In the Netherlands, anesthesiological care is arranged as a set fee for an episode of care related to a surgical procedure. This fee includes all (expected costs for) anesthesia, pain medicine and related services during and shortly after the surgical procedure, including potential complications and subsequent procedures [23]. However, this system complicates the accurate assessment of the actual costs incurred by this medical specialty. Consultations are not consistently reported, and the level of involvement varies per-patient. As a result, precise total costs cannot be fully derived from in-hospital resource data, unlike other medical specialties. Despite these challenges, the anesthesia related costs are likely to be relatively small compared to other healthcare costs and may have minimal impact on the overall cost-effectiveness evaluation.

The trial’s strengths, detailed in the primary paper, included its randomized controlled design across four high-volume centers, with a representative esophageal cancer patient population in the Western world, and quality control for paravertebral catheter insertions [10]. Additional strengths of the current analysis included the bottom-up approach for cost calculations, to capture all relevant cost components. Furthermore, in-hospital costs were derived from hospital registries, with complete data available for all but two patients and a detailed overview of healthcare resource use.

The limitations of the primary study, such as the lack of a specified imputation plan for the primary outcome, the use of continuous epidural infusion without patient-controlled bolus function, and the superiority trial design, have been previously described [11]. Specific to this cost-effectiveness analysis, additional limitations included the unavailability of certain specific costs that would have been valuable, such as those related to the involvement/consultation of the anesthesiologist, were not available, as previously mentioned.

Another limitation is the lack of out-of-hospital healthcare resource use and utility data. In the primary analysis of the PEPMEN-trial, the QoR-40 questionnaire on POD 3 was used as the primary outcome. POD 3 was chosen as the timing, anticipating the greatest impact of analgesia techniques in the early postoperative days. Patients in this trial were also asked to complete the EQ-5D-5L questionnaire at three and six months after surgery [19]. However, we anticipated that using these EQ-5D-5L measurements would more likely reflect the quality of recovery/life due to the esophageal cancer (treatment) rather than the effects of the investigated analgesia techniques. We, therefore, decided to use the QoR-40 measurements as the effectiveness measure in the cost-effectiveness analysis. This approach aligns our conclusions regarding effectiveness with those of the main article of the PEPMEN-trial and, while also highlighting that even when clinical outcomes are comparable, a detailed economic evaluation can provide valuable insights into cost differences and resource use.

In conclusion, cost differences related to materials, OR time, and medication use between epidural and paravertebral analgesia, were minimal compared to the total in-hospital costs, in both complicated and uncomplicated patients. Consequently, cost-effectiveness is mainly influenced by effectiveness, which was slightly lower for paravertebral analgesia. While paravertebral analgesia does not prove to be a cost-effective alternative compared to epidural analgesia, these findings support its consideration as a clinically viable option in patients undergoing MIE.

## Supplementary Information

Below is the link to the electronic supplementary material.

Supplementary file1 (DOCX 397 kb)
