# Supplementary material for: Cost-effectiveness of Paravertebral versus EPidural analgesia in Minimally invasive Esophageal resectioN (PEPMEN): an economic evaluation alongside a randomized clinical trial
Source: Surg Endosc. 2026 Mar 4;40(5):4056–67. doi: 10.1007/s00464-026-12642-7 (PMC13161283; doi:10.1007/s00464-026-12642-7)
Supplement: Supplementary file 1 — Supplementary file1 (DOCX 397 kb) [file 464_2026_12642_MOESM1_ESM.docx]

**SUPPLEMENTARY MATERIALS**

**Supplementary Figure S1:** Flowchart of patient inclusion in the PEPMEN trial


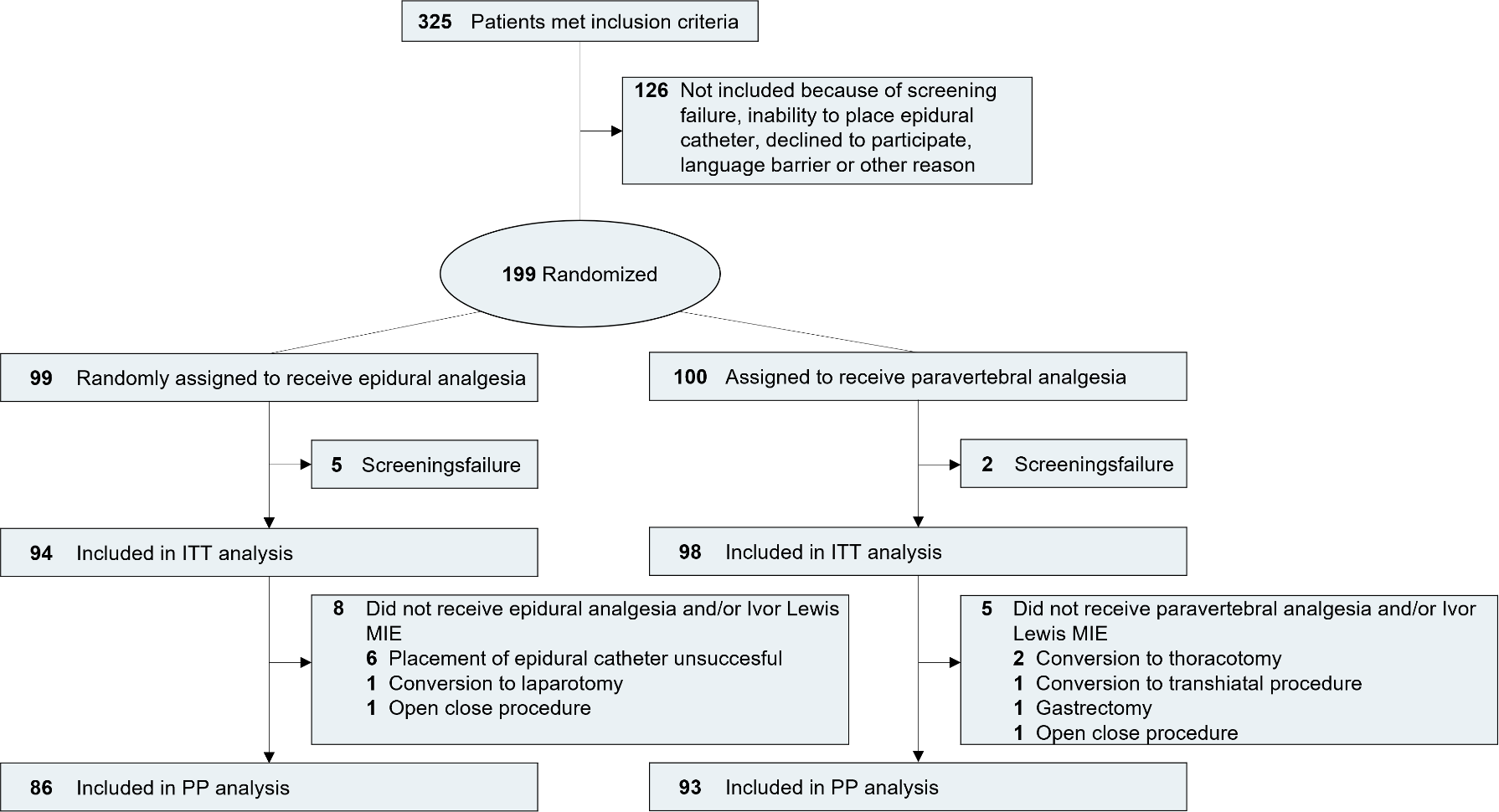


*All patients who underwent random assignment, without screening failure, (n=192) were included in the intention-to-treat analysis, 94 in the epidural analgesia group and 98 in the epidural analgesia group. A total of 179 patients underwent Ivor Lewis minimally invasive esophagectomy and their allocated analgesia modality according to the protocol, 86 in the epidural analgesia group and 93 in the paravertebral analgesia group.*

*Abbreviations: ITT = Intention to treat; PP = Per protocol.*

**Supplementary Table S1:** Laparoscopic equipment costs per surgical procedure

| **Product** | **Purchase costs, €** | **Product lifespan, years** | **Purchasing costs per year (depreciation included), €** | **Yearly costs for maintenance, €** | **Yearly costs (maintenance and depreciation included), €** | **Costs per business day, €** | **Costs per procedure, €** |
| --- | --- | --- | --- | --- | --- | --- | --- |
| *Laparoscope* | 12,266.00 | 10 | 1,226.60 | 1,839.90 | 3,066.50 | 25.55 | 25.55 |
| *Camera head* | 5,100.00 | 10 | 510.00 | 765.00 | 1,275.00 | 10.63 | 10.63 |
| *Light source* | 6,460.00 | 10 | 646.00 | 484.50 | 1,130.50 | 9.42 | 9.42 |
| *Video processor* | 9,075.00 | 10 | 907.50 | 680.63 | 1,588.13 | 13.23 | 13.23 |
| *Insufflator* | 5,339.00 | 10 | 533.90 | 400.43 | 934.33 | 7.79 | 7.79 |
| *Trolley* | 2,532.00 | 10 | 253.20 | 189.90 | 443.10 | 3.69 | 3.69 |
| *Two monitors* | 11,200.00 | 10 | 2,240.00 | 840.00 | 3,080.00 | 25.67 | 25.67 |
| *Monitor suspension system* | 4,283.00 | 10 | 428.30 | 321.23 | 749.53 | 6.25 | 6.25 |
| ***Total*** | 56,255.00 | NA | 6,745.50 | 5,521.58 | 12,267.08 | 102.23 | 102.23 |

*Based on eTable 2 of ‘Laparoscopic Versus Open Gastrectomy for Gastric Cancer (LOGICA): A Multicenter Randomized Clinical Trial – E van der Veen et al. JAMA Surgery. 2021’*

*Abbreviations: NA, Not applicable.*

Laparoscopic and thoracoscopic equipment purchasing costs were €56,255.00, as obtained by the purchase department of one of the participating hospitals. By dividing these costs by the product life span, purchasing costs per year were calculated to be €6,745.50. Based upon standard hospital policy, yearly maintenance costs are 15% of purchasing costs for the laparoscope and camera head and 7% of the purchasing costs for the other equipment, resulting in yearly maintenance costs of €5,521.58. Hence yearly costs to provide one operation room with laparoscopic equipment (including purchase costs, depreciation and maintenance) were €12,267. A year was assumed to contain 240 business days, of which the laparoscopic equipment was assumed to be used on 120 business days (either for minimally invasive esophagectomy or other laparoscopic surgery). A minimally invasive esophagectomy was assumed to take an entire business day. Hence costs per minimally invasive esophagectomy were estimated to be (€12,267 / 120 days) €102.23.

**Supplementary Table S2:** Disposable costs of the epidural and paravertebral disposable costs per center

|  | **Catharina** | **ZGT** | **UMC Utrecht** | **Amsterdam UMC** | ***Mean cost (total), €*** |
| --- | --- | --- | --- | --- | --- |
| ***Epidural (n=94)*** | €43.25 | €22.04 | €33.78 | €28.00 | €30.13 |
| ***Paravertebral (n=98)*** | €40.05 | €20.15 | €21.10 | €28.00 | €25.63 |

**Supplementary Figure S2:** Cost-effectiveness planes of sensitivity analysis with minute price of operation in Dutch guidelines

**
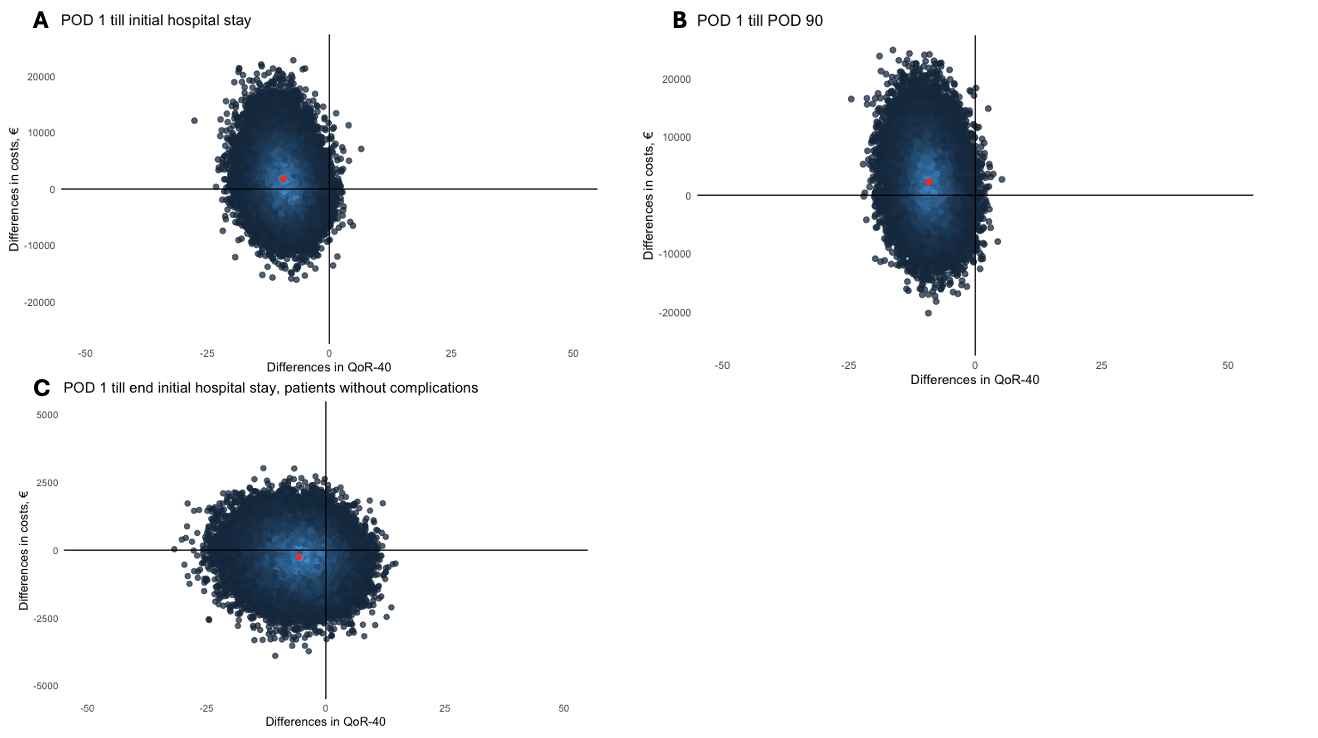
**

*Costs and QoR-40 scores for the epidural group compared to the paravertebral group across 2,000 bootstrap iterations are shown in cost-effectiveness planes. Costs of minute price of operation room are adjusted to €11,09 in this analysis based on the guideline for health economic evaluation in the Netherlands [13] instead of €22,00 based on the research of Bolkenstein et al. [16] in the main analysis. (A) Cost-effectiveness plane for the initial hospital stay. (B) Cost-effectiveness plane from postoperative day 1 to 3 months post-surgery. (C) Cost-effectiveness plane for the initial hospital stay, including only patients without complications. Each dot represents the difference in cost and QoR-40 score between epidural and paravertebral analgesia for a single bootstrap iteration. Iterations in the upper left quadrant indicate higher costs and lower QoR-40 scores for the paravertebral group, the upper right quadrant shows higher costs and higher QoR-40 scores, the lower right quadrant represents lower costs and higher QoR-40 scores, and the lower left quadrant shows lower costs and lower QoR-40 scores for the paravertebral group. The shade of blue indicates overlapping dots, with light blue representing the highest density.*

Changing the costs of minute price of operation to €11,09 based on the guideline minimally affected the spread of the iterations over the quadrants in all scenarios. In the scenarios including all patients until the end of initial hospital stay, the mean remains in the upper left quadrant. Most iterations are located in this quadrant 62.9%, followed by 36.8% in the bottom left. 0.2% of the iterations is located in the upper right quadrant and 0.2% is located in the bottom right quadrant. A small shift is seen from the bottom left to the upper left quadrant, meaning that with the guideline minute price paravertebral has slightly more iterations with higher costs and lower QoR-40 scores.

In the second scenario, including all patients until POD 90. 64.9% of the iterations are located in the upper left quadrant and 34.8% in the bottom left quadrant. 0.2% of the iterations is located in the upper right and 0.2% in the bottom right. Again, a small shift is seen from the bottom left to the upper left quadrant.

In the third scenario only patients without complications during initial hospital stay are included. The iterations are still primarily spread over the left quadrants, with 32.9% in the upper left and 51.4% in the bottom left, 10.7% in the bottom right and 5.0% in the upper right. In this scenario the change in cost of minute price of operation affected the spread in all four quadrants. However, the mean remains in the bottom left quadrant (Figure S3).
